# Technology Transfer of the Microphysiological Systems: A Case Study of the Human Proximal Tubule Tissue Chip

**DOI:** 10.1038/s41598-018-33099-2

**Published:** 2018-10-05

**Authors:** Courtney Sakolish, Elijah J. Weber, Edward J. Kelly, Jonathan Himmelfarb, Roula Mouneimne, Fabian A. Grimm, John S. House, Terry Wade, Arum Han, Weihsueh A. Chiu, Ivan Rusyn

**Affiliations:** 1 Department of Veterinary Integrative Biosciences, Texas A&M University, College Station, TX USA; 2 Department of Pharmaceutics, University of Washington, Seattle, WA USA; 3 Division of Nephrology, University of Washington Kidney Research Institute, Seattle, WA USA; 4 Bioinformatics Research Center, North Carolina State University, Raleigh, NC USA; 5 Geochemical and Environmental Research Group, Texas A&M University, College Station, TX USA; 6 Department of Electrical and Computer Engineering, Texas A&M University, College Station, TX USA; 7 Department of Biomedical Engineering, Texas A&M University, College Station, TX USA

## Abstract

The adoption of a new technology into basic research, and industrial and clinical settings requires rigorous testing to build confidence in the reproducibility, reliability, robustness, and relevance of these models. Tissue chips are promising new technology, they have the potential to serve as a valuable tool in biomedical research, as well as pharmaceutical development with regards to testing for efficacy and safety. The principal goals of this study were to validate a previously established proximal tubule tissue chip model in an independent laboratory and to extend its utility to testing of nephrotoxic compounds. Here, we evaluated critical endpoints from the tissue chip developer laboratory, focusing on biological relevance (long-term viability, baseline protein and gene expression, ammoniagenesis, and vitamin D metabolism), and toxicity biomarkers. Tissue chip experiments were conducted in parallel with traditional 2D culture conditions using two different renal proximal tubule epithelial cell sources. The results of these studies were then compared to the findings reported by the tissue chip developers. While the overall transferability of this advanced tissue chip platform was a success, the reproducibility with the original report was greatly dependent on the cell source. This study demonstrates critical importance of developing microphysiological platforms using renewable cell sources.

## Introduction

Microphysiological systems (MPS) have gained attention due to their unique abilities to recapitulate the dynamic microenvironment and structure of cells and tissues^1^. Many studies demonstrated that MPS can maintain physiological cell function and behaviour, as well as recapitulate the induction and propagation of diseased states and toxicities^2–8^. These state of the art *in vitro* models have the ability to significantly impact the process of drug development; however, adoption of MPS by the industry and regulatory agencies has been slow^9,10^. In addition to a concern about low reproducibility rates within life science research^11^, a common challenge to wider use of MPS has been a lack of confidence in the reliability and relevance of these models outside of their developer’s laboratories. This gap is being addressed by the National Center for Advancing Translation Science through independent testing of the robustness and reproducibility of the MPS^9^.

To build confidence in the utility of the MPS, a strategic roadmap to bridge the gap between the innovators and end users through independent testing process has been proposed^9,10^. One example of such a process is the guidance on validation and international acceptance of new or updated test methods^12^; however, challenges with applying this guidance to rapidly developing technologies have been well documented^13^. The National Academies Report^13^ recommended a focus on establishing the reliability of the new test methods, defining the domain of applicability for these technologies, describing how test results should be interpreted in terms of a positive/negative response, and development of performance standards for evaluation of relevant adverse outcomes. Thus, the study detailed herein followed the recommendations from the National Academies^13^, the industry^10^, and the government^9^ and conducted a cross-laboratory testing of reliability and relevance of a recently developed proximal kidney tubule MPS^14,15^.

The proximal tubule has been a focus for tissue chip developers because of its importance in the physiological function of the kidney and in drug and chemical disposition^15,16^. The MPS that was selected for testing^14,15^ is a human proximal tubule tissue chip that was shown to replicate the polarity of the tubule and express key proteins (aquaporin 1, GGT, and SGLT2). Renal proximal tubule epithelial cells (RPTECs) self-assemble into a 3D structure in the device, maintain viability, morphology and function for up to 24 days under laminar flow of the media, and demonstrate metabolic competence.

In this work, we aimed to identify the challenges with technology transfer of MPS by replicating key prior observations from the proximal kidney tubule MPS^14,15^, as well as used this model to study nephrotoxicity of several reference compounds (polymyxin B, cisplatin, gentamicin, and cadmium). Endpoints were selected from the developer’s previously published work. Studies in the proximal kidney tubule MPS were carried out in parallel with traditional 2D cultures of RPTECs to determine when 3D models exceed traditional *in vitro* cultures. Finally, to test the impact of the source of human cells, we used two different cell sources for human RPTECs, one was from the MPS developer lab, and another one was from a commercial supplier. The experiments were focused on the ability to maintain long-term functionality of the MPS, and investigation of the effects of classical nephrotoxicants. Phenotyping assays ranged from phase-contrast and fluorescent imaging to analytical chemistry, analysis of the effluent for toxicity markers, and gene expression profiling. This manuscript details the results of the first NIH-funded study that focused on the investigation of tissue chip technology transfer and reproducibility of the data outside of the developer laboratory through a case study of independent testing of the proximal tubule chip. We focused on the transferability, repeatability, and relevance of the model for future testing of drugs and chemicals.

## Materials and Methods

### Cell culture in 2D and 3D platforms

Primary human kidney cells (designated as “HIM-31 cells” throughout this manuscript) were obtained from the University of Washington. Cells were isolated and propagated from healthy kidney tissue as described previously^17^, and cryopreserved prior to shipment. Upon receipt at Texas A&M University, these cells were cultured at 37 °C and 5% CO_2_ in PTEC media [DMEM/F12 media (Gibco, Waltham, MA), supplemented with ITS-A (Sigma-Aldrich, St. Louis, MO), hydrocortisone (Sigma-Aldrich), and antibiotic-antimycotic supplement (ThermoFisher, Waltham, MA)]. As an additional cell source, human renal proximal tubule epithelial cells (RPTECs) were obtained from Lonza (CC-2553, Lot #0000581945; Basel, Switzerland). These cells (designated as “Lonza cells” throughout this manuscript) were cultured at 37 °C and 5% CO_2_ in renal epithelial growth medium (REGM, Lonza) that was supplemented with fetal bovine serum (0.5%), human transferrin (10 mg/mL), hydrocortisone (0.5 mg/mL), insulin (5 mg/mL), triiodothyronine (5 × 10^−12^ M), epinephrine (0.5 mg/mL), epidermal growth factor (10 mg/mL), and antibiotics (100 U/mL penicillin and 100 mg/mL streptomycin). Cells between passages 2–4 (HIM-31) or 4–6 (Lonza) were used in the subsequent 2D and 3D studies.

The microfluidic platform used in these studies was from Nortis Bio (Seattle, WA)^15^. This polydimethylsiloxane (PDMS) and glass device contained a single fluidic channel, as well as ports to fill a central growth area that contains a microfiber filament insert. The device preparation and cell seeding protocols were adapted from previous work by the developers^15^. Briefly, the growth area of the chip was filled with collagen type I (6 mg/mL; Ibidi, Martinsried, Germany), and the matrix was allowed to polymerize around the microfiber filament for 24 hours at room temperature. After polymerization, the filament insert was carefully removed from each device to form a suspended luminal channel within the gelled collagen I extracellular matrix, connected to the fluidic channel, allowing for fluid flow through the lumen. This newly formed channel was then coated with collagen type IV (5 μg/mL; Corning, Corning, NY) for 30 minutes at 37 °C and either PTEC media (HIM-31 cells) or REGM (Lonza cells) was flushed through the system to remove excess collagen IV at a rate of 2 μL/min for 1 hour.

Confluent monolayer cultures of proximal tubule cells of both sources were treated with 0.05% Trypsin-EDTA (ethylenediamine tetraacetic acid) to detach from plates, stained with Trypan Blue, and counted. Cell pellets were resuspended to a concentration of 20 × 10^6^ cells/mL, and 3.5 μL of this suspension was injected into the lumen of each device. Cells were allowed to adhere for 24 hours prior to the introduction of physiologic fluid flow at 0.5 μL/min with corresponding media. This flow rate resulted in cells being exposed to ~0.25 dyne/cm based on the equation for fluid shear stress, τ = 6Qµ/bh2 (where τ is the shear stress at the surface of the cells [dyne/cm^2^], Q was the flow rate [8.3 × 10^−6^ cm^3^/s], µ was the viscosity of the culture media [8.9 × 10^−3^ dyn*s/cm^2^], b was the width of the channel [0.012 cm], and h was the height [0.012 cm]). Devices were maintained under constant flow for up to 24 days. In parallel, 384-well plates were coated with collagen IV as described above and RPTECs were seeded at a density of 100,000 cells/cm^2^ in corresponding media. Media in 384-well plates was exchanged every 3 days, and cells were maintained for up to 21 days after initial seeding.

Chemical treatments were performed on cells that were grown in plates or devices (0.5 μL/min flow rate) for 1 week. Culture media containing polymyxin B (final concentration of 50 μM; X-Gen Pharmaceuticals; Horseheads, NY), cisplatin (6.4 and 64 μM; SigmaAldrich), gentamicin (200 and 600 μM; Hospira UK, Hurley, UK), or cadmium chloride (0.05 and 0.5 μM; SigmaAldrich) was exchanged every 3 days, and effluent samples were collected throughout the exposure period.

### Cell viability

Throughout the experiment, cell viability was monitored by phase-contrast microscopy (integrity of the cell lining of the channel’s lumen), as well as by measuring KIM-1 and LDH in the perfusate collected daily using ELISA (KIM-1) or biochemical (LDH) assays (R&D Systems, Minneapolis, MN) following manufacturer’s protocols. At each time-point in the experiment, cell viability in MPS devices and 384-well plates was assessed using LIVE/DEAD Viability/Cytotoxicity assay (Invitrogen, Carlsbad, CA).

### Immunocytochemistry

Cells in the devices and on 384-well plates were fixed with 10% buffered formalin-acetate for 1 hour. After fixation, cells were permeabilized with 0.5% Triton X-100 (ThermoFisher), and blocked with 0.1% bovine serum albumin (Sigma-Aldrich) for 1 hour. Cells were rinsed with physiologically-buffered saline (PBS), and primary antibodies (anti-SLC22A11/OAT-4 (Abcam, 1:100), and anti-Heme Oxygenase-1 (Abcam, 1:500), were added and allowed to incubate overnight at 4 °C. Cells were rinsed again with PBS and secondary antibodies anti-rabbit IgG conjugated Alexa Fluor® 488 (Abcam, 1:100) and anti-mouse IgG conjugated Alexa Fluor® 647 (LifeTechnologies, 1:100) were applied and allowed to incubate for 1 hour at 37 °C. Cells were rinsed again with PBS, and mounted using Prolong® Gold antifade with DAPI (Invitrogen). Plates were imaged using an ImageXpress Micro Confocal High-Content Imaging System (Molecular Devices, Sunnyvale, CA) and devices were imaged with LSM 780 NLO Multiphoton Microscope (ZEISS, Oberkochen, Germany).

### Ammoniagenesis assay

Cells were grown over 2 weeks (>75% confluency) in the devices or on plates, and were evaluated for their ability to respond to pH changes via monitoring of ammonia secretion. Cells were perfused/cultured in PBS (pH 7.4) for 4 hours (1 μL/min flow rate within devices to allow for a sufficient effluent volume for testing over this short exposure period, corresponding to 0.51 dyne/cm^2^ shear stress on the cell surface), followed by PBS (pH 6.9) for another 4 hours. Effluent and cells were collected at the end of each treatment, and tested for ammonia concentration using an ammonia assay kit (Abcam).

### **V**itamin D metabolism

Cells were grown for 3 weeks within devices or plates, and were assessed for their ability to respond to the presence of 1α, 25-(OH)_2_ vitamin D3 (calcitriol, Toronto Research Chemicals, Toronto, Canada) via induction of the 24-hydroxylation metabolic pathway for conversion of 25-OH vitamin D3 (calcidiol, Toronto Research Chemicals) into 24,25 dihydroxy vitamin D3. Cells were exposed to 1 μM calcidiol-containing media (0.5 μL/min perfusion in devices) in the presence or absence of 0.5uM exogenous calcitriol. Effluent samples were collected every 24 hours over 3 days of exposure, and stored at **−**80 °C until analysis of calcidiol and 24,25-(OH)_2_ vitamin D3 concentration as detailed elsewhere^14^.

### **G**ene expression profiling

Transcriptomic analysis of kidney proximal tubule cells was conducted using the Templated Oligonucleotide Sequencing Assay (TempO-Seq™, BioSpyder Technologies, Carlsbad, CA) as detailed elsewhere^18,19^. Briefly, cell lysates from tissue chips were prepared through injection of 30 µl lysis buffer into the luminal channel. Following 10 minutes of incubation at room temperature, the cell lysate was flushed out with an estimated cell content of ~150/µL. For 384 well plates, media was aspirated and 10 µl of lysis buffer was added and cells were incubated for 10 minutes at room temperature. All lysate samples were stored at **−**80 °C until testing. TempO-seq libraries were prepared using the ToxPanel targeted transcriptome panel consisting of 2,982 transcripts according to the manufacturer’s instructions. Briefly, hybridization of the mRNA content was achieved by incubation of 2 µl cell lysate and 2 µl hybridization mix as follows: 10 min at 70 °C, cooling ramp from 70 °C to 45 °C over 49 min, 1 min at 45 °C. Excess oligonucleotides were then digested in a nuclease catalyzed reaction for 90 min at 37 °C. Next, hybridization products were incubated with DNA ligase for 60 min at 37 °C. Nuclease and ligase were then denatured using a heat denaturation step (80 °C for 30 min). 10 µl of each ligation product was then mixed with an equal volume of PCR amplification mix and amplified in a LightCycler 96 (Roche, Basel, Switzerland) using the manufacturer recommended settings. PCR primers contained unique, oligonucleotide sequence-encoded sample barcodes, allowing unambiguous computational sample identification. Amplicon samples (5 µl) were then pooled and purified using a commercial PCR clean-up kit (Clontech, Mountain View, CA). Pooled libraries were sequenced in 50 single-end read mode using a rapid flow cell on a HiSeq 2500 Ultra-High-Throughput Sequencing System (Illumina, San Diego, CA).

### **A**nalytical chemistry

The bioanalysis of 25-OH vitamin D3, 24,25 -(OH)_2_ vitamin D3, and 1α,25-(OH)2 was performed at the University of Washington site via a previously established^20^ LC-MS/MS method with modifications that are listed in the SI methods as in Weber *et al*.^14^. Briefly, 250 µL aliquots of perfusate from devices, or pooled cell culture media from wells were measured on a Nexera X2 LC-30AD UHPLC (Shimadzu, Kyoto, Japan) coupled to an AB/SCIEX QTRAP 6500 mass spectrometer (Sciex, Framingham, MA) to determine the concentrations of 25-OH Vitamin D3 and its metabolites in samples. The formation clearance (CL_f_) for 1α,25-(OH)_2_ vitamin D3 and 24,25-(OH)_2_ vitamin D3) was plotted over time. The method for calculating CL_f_ is described in Weber *et al*.^14^.

### Availability of materials and data

All data reported herein are available from the University of Pittsburgh Drug Discovery Institute Microphysiology Systems Database (https://mps.csb.pitt.edu/).

## Results

### Long-term viability and functionality of RPTECs in 3D MPS and 2D culture

**Figure 1:**
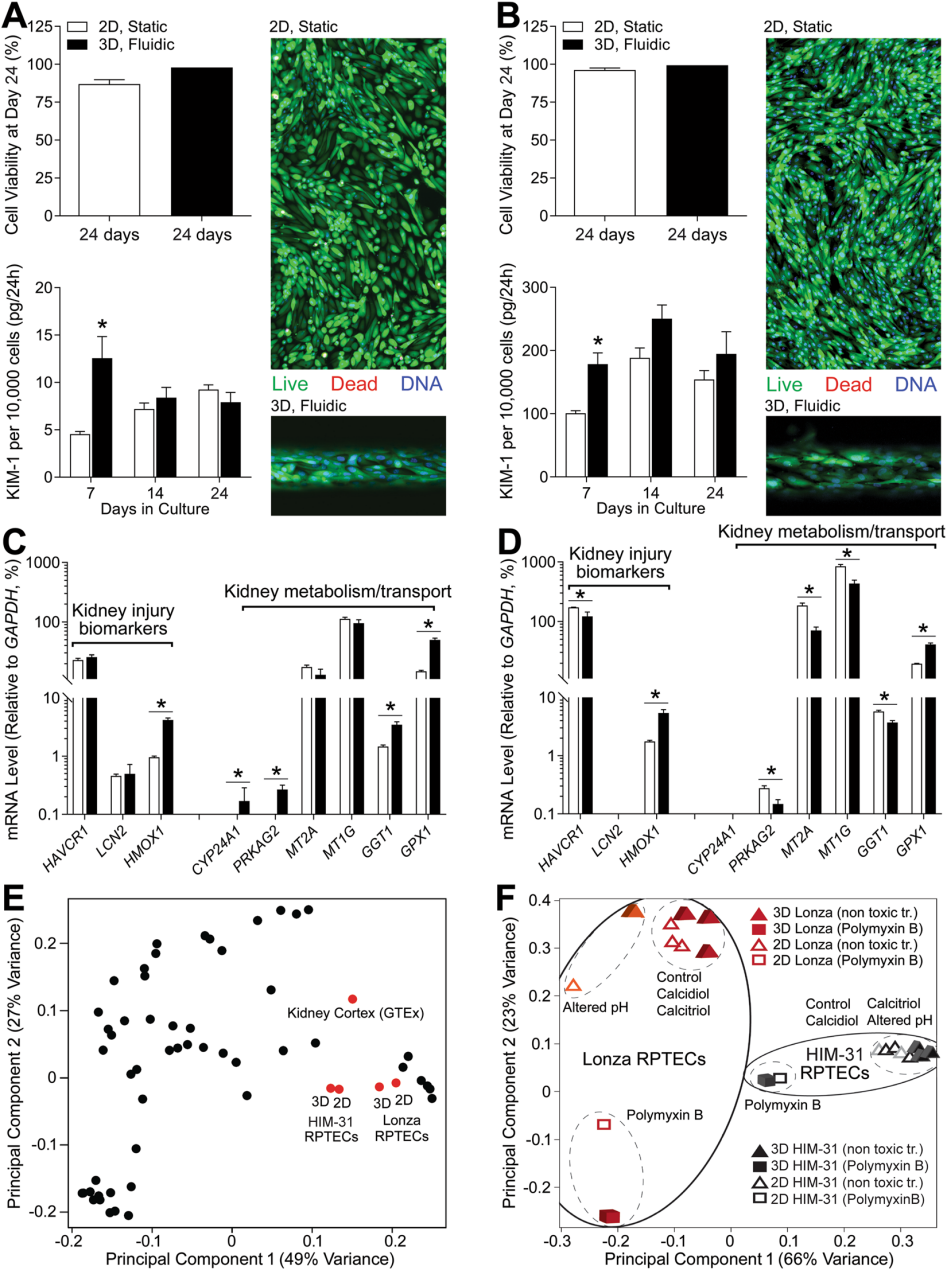
Long-term culture of RPTEC under 3D and 2D conditions. Cell viability and KIM-1 secretion in 2D and 3D (**A**) HIM-31 and (**B**) Lonza RPTEC cultures. Baseline gene expression in (**C**) HIM-31 and (**D**) Lonza RPTEC cultures, highlighting differences between 2D and 3D culture in expression of kidney injury biomarkers, and metabolism and transport markers. (**E**) Principal component analysis demonstrating grouping between cell sources and culturing conditions in comparison to GTEx human kidney cortex tissues. Black data points represent other human tissues within GTEx. (**F**) Principal component analysis of 2D and 3D cell cultures, demonstrating grouping within cell source, and toxic (polymyxin B) vs non-toxic (controls, calcidiol, calcitriol, and altered pH) exposure conditions. (*p < 0.05, 2-tailed t-test).

HIM-31 and Lonza cells were cultured in 2D (384-well plates) or in 3D (MPS under flow of 0.5 μL/min) over a period of 24 days. Cell media or perfusate were collected at 1-week intervals and at 24 days after seeding cells were stained with CalceinAM and ethidium bromide to determine viability (Fig. 1A,B). Excellent viability was observed in both 2D and 3D culture conditions for either HIM-31 cells (Fig. 1A, 2D n = 5; 3D n = 1), or Lonza cells (Fig. 1B, 2D n = 11; 3D n = 1); however, there were clear differences in cell morphology between 2D and 3D cultures. Both cell types that were grown in 3D under shear stress self-organized, elongated, and aligned in the direction of fluid flow, while cells grown in 2D lacked this organization.

In addition, shedding of KIM-1 from RPTECs was evaluated throughout the growth period (on days 7, 14, and 24). In both cell types the patterns of KIM-1 changes over time or differences between 2D (n ≥ 12 HIM-31, n ≥ 5 Lonza) and 3D were similar. KIM-1 release on day 7 was about 2-fold higher in 3D cultures compared to 2D static culturing conditions; however, KIM-1 release increased and converged between 2D and 3D conditions by day 14. The most pronounced difference in KIM-1 release was observed between cell types, at the basal level HIM-31 cells released 10–20 times less KIM-1 than Lonza cells.

Next, we evaluated gene expression profiles in RPTECs cultured in 2D and 3D for 24 days (Fig. 1C,D). Expression of representative genes for kidney injury markers^21^ and kidney metabolism/transport is shown. Basal expression of *KIM-1* (also known as *HAVCR1*) was about 10-fold lower in HIM-31 cells (Fig. 1C) then in Lonza cells (Fig. 1D), consistent with the KIM-1 measurements in the perfusate. Heme oxygenase 1 (*HMOX1*) expression was significantly higher in 3D cultures, but did not differ between HIM-31 and Lonza cells. Another toxicity marker, neutrophil gelatinase-associated lipocalin (*LCN2*; also known as *NGAL*) was expressed at very low levels or undetectable in both cell types under basal conditions. Likewise, inducible genes, like *CYP24A1*, were undetectable or low expressed in both cell types. On the other hand, expression of metallothioneins, gamma-glutamyl transferase and glutathione peroxidase 1, genes that are highly constitutively expressed in renal proximal tubule, was robust in both cell types with some differences between 2D and 3D culture conditions. Overall, the transcriptome of RPTECs of both cell sources that were cultured in either 2D or 3D was largely similar (Fig. 1E, no difference in the first principal component), and consistent to that of the human kidney cortex samples profiled by the human Genotype-Tissue Expression (GTEx) project^22,23^. However, when gene expression profiles were compared between HIM-31 and Lonza cells, in both basal expression and various culture and challenged conditions (Fig. 1F), two cell types and 2D and 3D culture conditions were factors that showed most separation in the transcriptional state.

**Figure 2:**
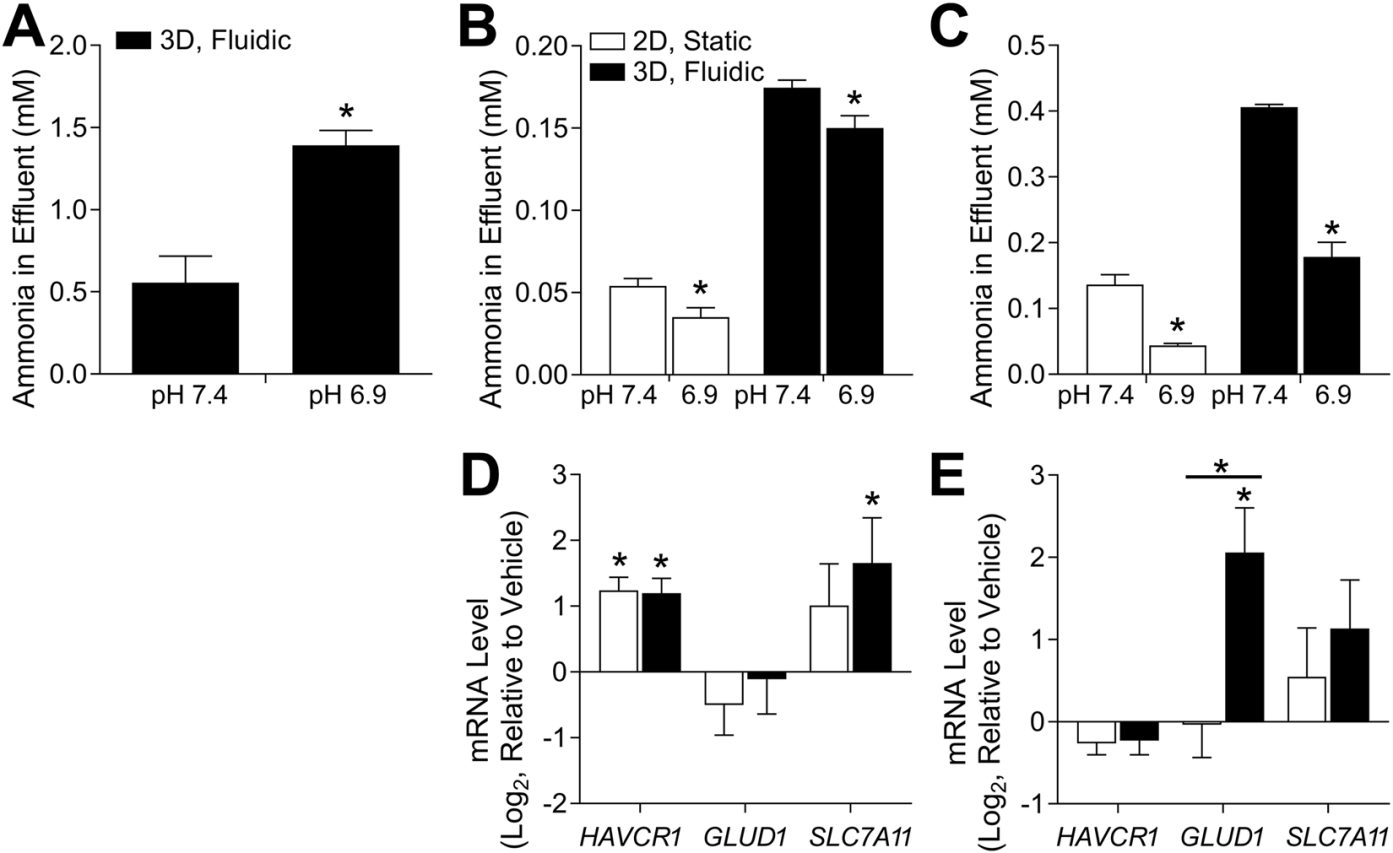
Ammoniagenesis in response to altered pH. Initially, media was at a pH of 7.4, and was changed to 6.9 to determine differences in ammonia secretion between conditions. Ammonia concentration in effluent is shown in (**A**) the previous publication13, (**B**) HIM-31, and (**C**) Lonza RPTECs. Additionally, expression of select genes is shown in (**D**) HIM-31, and (**E**) Lonza RPTECs as log2fc relative to vehicle. (*Differentially expressed gene compared to vehicle, p < 0.05, 2-tailed t-test).

The developers of the MPS^14^ that was tested herein demonstrated that when human primary RPTECs, a cell line different from those tested in our study, were subjected to less than physiological pH within the MPS, they responded with a 3-fold increase in excretion of ammonia (Fig. 2A, HIM-31 n ≥ 6; Lonza n ≥ 8). This experiment was repeated herein; however, increased ammonia secretion was not observed after altering pH of the culturing conditions, regardless of the cell type or culture conditions (Fig. 2B,C). In fact, ammoniagenesis decreased in both cell types and culture conditions when the pH was lowered. Overall, the effect of lowered pH on the transcriptome of HIM-31 and Lonza cells was minimal (Fig. 1F). Only a few genes showed significant responses. For example, in response to changes in pH, HIM-31 cells showed increased expression of *HAVCR1* and *SLC7A11*, with no significant differences between culture conditions (Fig. 2D). *SLC7A11* has been shown to be involved in glutamate transport, an essential requirement for glutamaine metabolism, and therefore ammoniagenesis^24^. Additionally, excretion of *HAVCR1* was monitored across all culturing conditions as a “baseline” measurement for injury or stress to the tubular culture. Transcriptional effects of lower pH were different in Lonza cells (Fig. 2E), whereby no significant effect on either *HAVCR1* or *SLC7A11* was found, but a significant effect on expression of *GLUD1* was observed in 3D, but not 2D, cultures. This could be indicative of the capacity of these cells for ammoniagenesis, as GLUD1 is primarily responsible for glutamine metabolism^25^.

**Figure 3:**
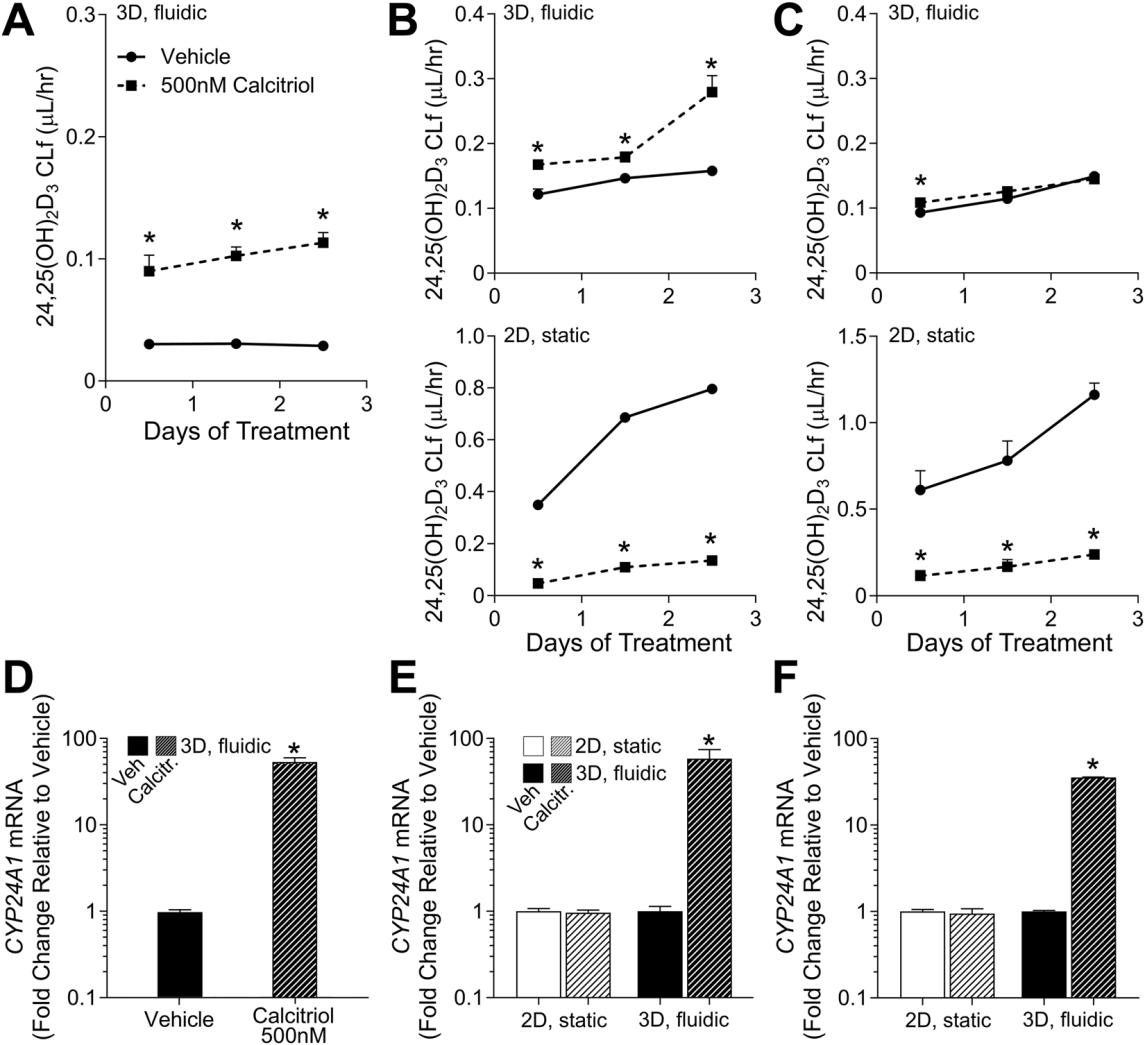
Metabolism of Vitamin D3 in 2D and 3D cultures. Formation clearance of 24,25(OH)_2_-Vitamin D3 after calcitriol exposure in (**A**) the previous publication13, (**B**) HIM-31, and (**C**) Lonza RPTECs. In the case of the HIM-31 and Lonza RPTECS, formation clearance was compared in 2D and 3D cultures. While a slight induction was observed in 3D HIM-31 cultures, no additional formation was observed in 3D Lonza cultures, and an unexpectedly negative effect was seen in all 2D cultures. Effect of calcitriol on CYP24A1 expression in (**D**) the previous publication13, and 2D and 3D (**E**) HIM-31 and (**F**) Lonza RPTEC cultures after 72 hours of exposure. Expression was normalized to that of GAPDH. (*Indicates significant change in expression relative to vehicle, p < 0.05 2-tailed t-test).

Another important physiological response of RPTECs demonstrated by the developers of this MPS^14^ was shifts in vitamin D metabolism in response to changes in co-factors and concomitant induction of *CYP24A1* (Fig. 3A,D). In this study, HIM-31 and Lonza cells were cultured within the MPS (n = 3), as well as in 2D culture (pooled sample from n = 5 wells), for 24 days and then exposed to either media containing only calcidiol (1 μM, vehicle), or both calcidiol (1 μM) and calcitriol (0.5 μM). We observed an effect on formation of 24,25-(OH)_2_ vitamin D3 in response to treatment with calcitriol in 3D HIM-31 cells (Fig. 3C), a regulatory feedback response similar to that reported in Weber *et al*.^14^. However, no effect was observed in 3D Lonza cultures (Fig. 3D). When 2D cultures of either HIM-31 or Lonza cells were treated with calcitrol, rather than seeing an increase in the formation of 24,25-(OH)_2_ vitamin D3, a significant decrease in net formation was observed (Fig. 3C,D). Additionally, *CYP24A1* expression was quantified in both cell types and culture conditions. We found that *CYP24A1* was induced 30–50 fold upon treatment with calcitriol in both HIM-31 and Lonza cells cultured in 3D, but not in either cell type cultured in 2D (Fig. 3E,F).

### Polymyxin B toxicity in RPTECs in 3D MPS and 2D culture

**Figure 4:**
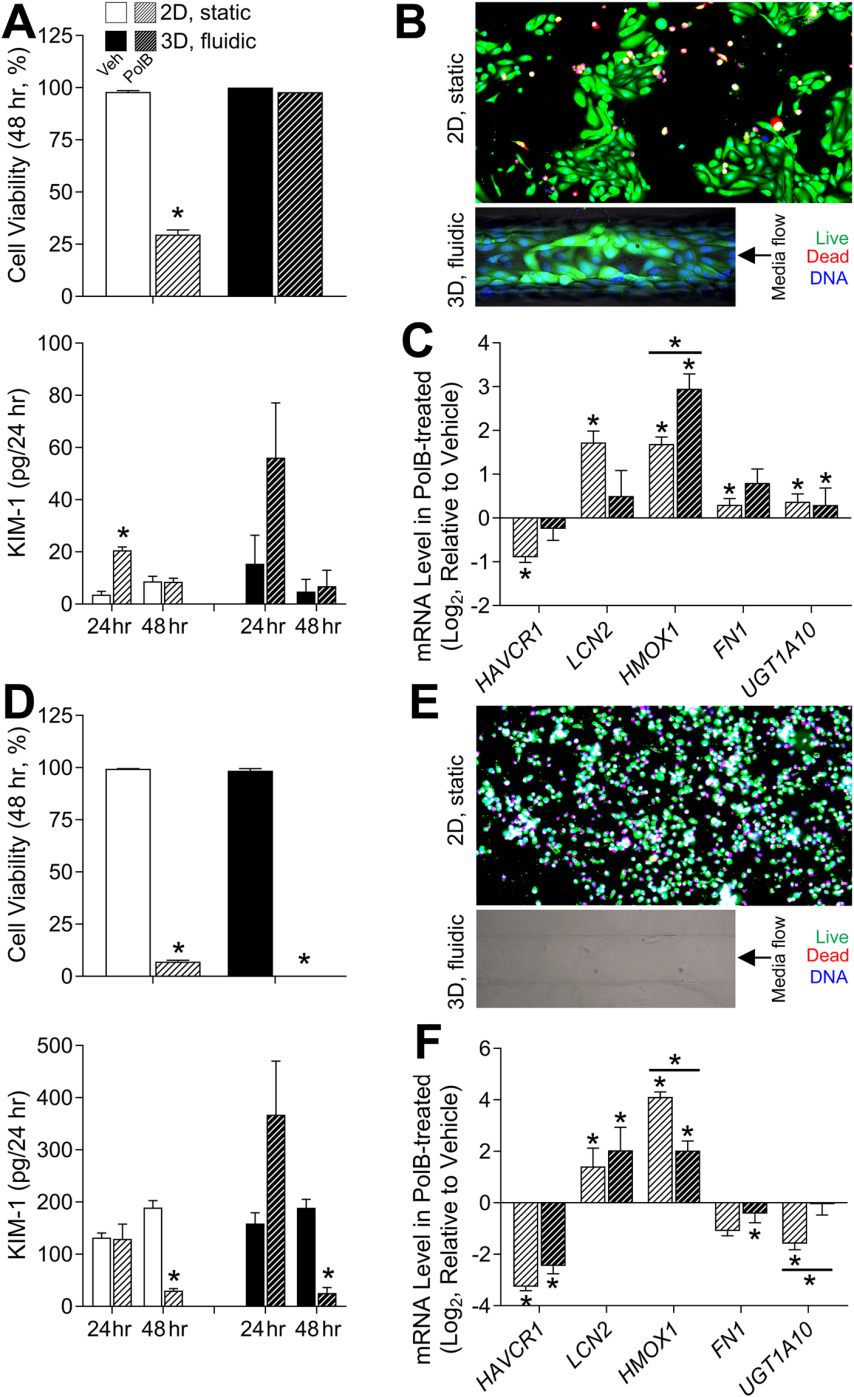
Polymyxin B toxicity in 2D and 3D cultures of HIM-31 (**A**–**C**) and Lonza (**D**–**F**) RPTECs. **A** and **D**: Cell viability and KIM-1 secretion in vehicle (solid white or black) and polymyxin B (50 μM, hashed white or black) treated (24 and 48 hrs) HIM-31(**A**) and Lonza (**D**) RPTECs in 2D (white) and 3D (black) cultures. Asterisks (*) denote statistical difference (p < 0.05, 2-tailed t-test) between groups. B and E: Representative images of calcein (green), ethidium (red), and Hoechst (blue) fluorescent staining in HIM-31(**B**) and Lonza (**E**) RPTECs in 2D and 3D that were exposed to polymyxin B (50 μM) for 48 hrs. **C** and **F**: Expression of genes involved in acute kidney injury was quantified in HIM-31 (**C**) and Lonza (**F**) RPTECs in 2D and 3D. Differentially expressed genes (relative to vehicle for each condition) are denoted with an asterisk (*), and significant differences between 2D and 3D cultures are denoted with an asterisk (*) and a bar.

Nephrotoxicity is a common adverse effect of the clinically used polymyxin-like antibiotics^26^. The adverse effects of polymyxin B were demonstrated in the proximal tubule MPS by the developers^27^. In this study, HIM-31 and Lonza cell cultures were grown in both 2D and 3D for 1 week and then exposed to polymyxin B (50 μM) for 48 hours. Effects of polymyxin B varied between cell source and culture condition (Fig. 4). In HIM-31 cells (Fig. 4A,B), marked cytotoxicity of polymyxin B was evident in 2D, but not 3D cultures. Interestingly, KIM-1 release into cell culture media or the perfusate was increased in the first 24 hrs upon exposure, but not at 48 hrs. This effect was observed in both 2D and 3D, albeit due to lower variability and greater number of replicates (3D n ≥ 2; 2D n ≥ 4), this effect was significant in 2D condition only. In their previous work Weber *et al*.^27^ noted a 4-fold increase in KIM-1 expression after polymyxinB exposure at 50 uM, which is highly comparable to the results of this study (3.7-fold increase). In Lonza cells (Fig. 4D,E), polymyxin B was markedly cytotoxic in both 2D (n = 8) and 3D (n ≥ 4), effectively emptying the lumen of the MPS devices due to cell death and media flow removing the debris. Significant effects on KIM-1 secretion were found only in treated cells at 48 hrs, but the effect was the opposite to the traditional use of this biomarker – the levels were low due to complete cell loss. In is noteworthy that basal levels of KIM-1 shedding into the media differed markedly between the cell types, the results that were similar to the observations shown in Fig. 1.

In addition, we examined the effects of exposure to polymyxin B on gene expression (Fig. 4C,F). In HIM-31 cells (Fig. 4C), significant induction of kidney injury genes and phase II metabolism genes was observed, in both 2D and 3D cultures, albeit the effect was more pronounced for the injury biomarkers in 2D cultures. This shows that even though cell viability was not impacted by polymyxin B in 3D cultures of HIM-31 cells, the adverse effect was discernible on the molecular level. In Lonza cells (Fig. 4F), marked induction of the injury markers was evident in both 2D and 3D; however, expression of fibronectin (*FN1*), and UDP glucuronosyltransferase (*UGT1A10*) was decreased as compared to HIM-31 cells.

### Studies of additional nephrotoxic compounds in RPTECs in 3D MPS and 2D culture

Exposure to many drugs and environmental chemicals has been associated with nephrotoxicity^28^. To extend the knowledge of the potential domain of applicability of the proximal tubule MPS to other compounds that may cause acute or chronic kidney effects, we performed additional studies with cisplatin (6.4 and 64 μM), gentamicin (200 and 600 μM), or cadmium (0.05 and 0.5 μM). These experiments were conducted using Lonza cells only due to limited availability of HIM-31 cells, that are not a renewable cell source from a single donor. Lonza cells were cultured in both 2D and 3D conditions. After the initial period of 7 days without any exposure, test compounds were added to cell culture media and treatments continued for up to 21 additional days.

**Figure 5:**
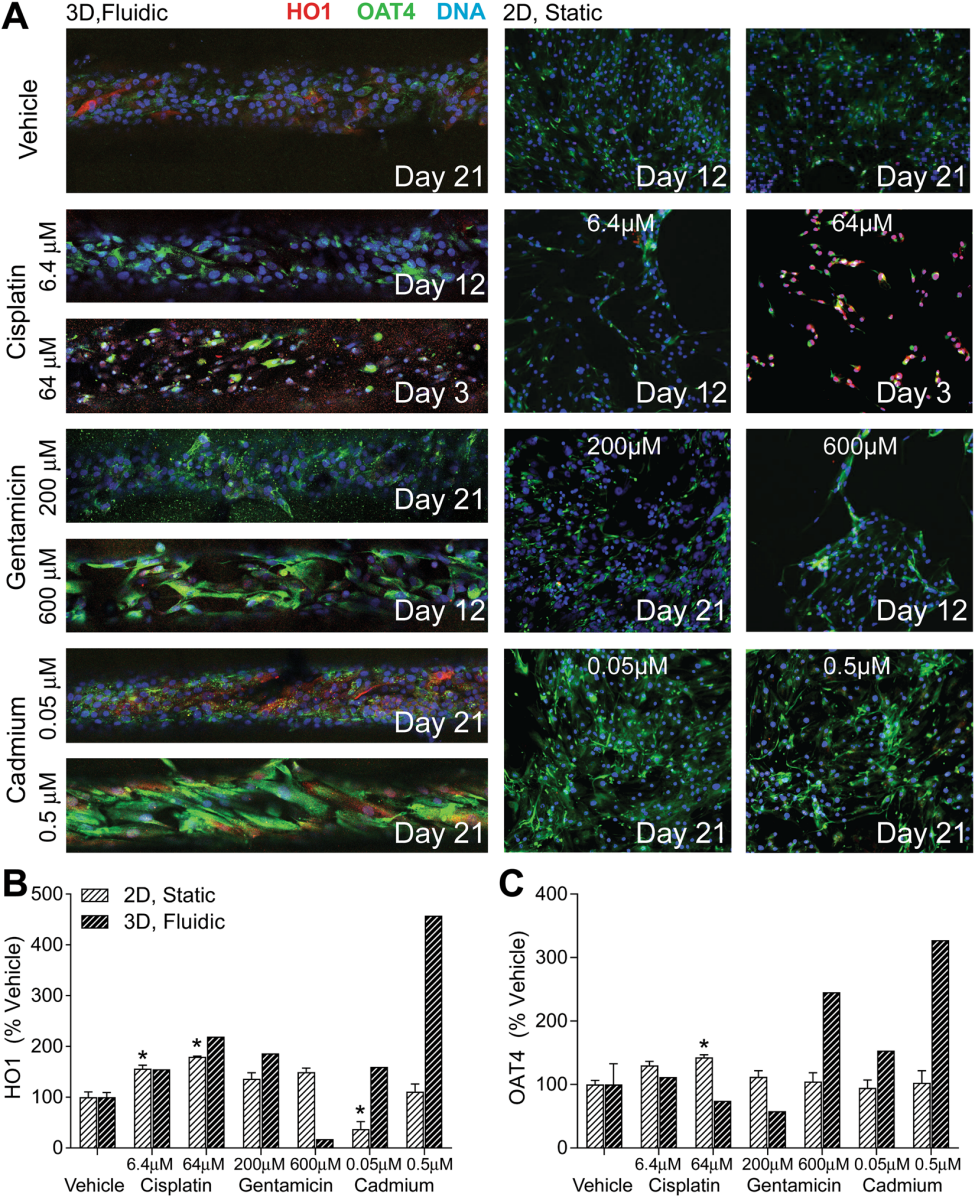
Effect of selected neprotoxicants on 2D and 3D Lonza RPTEC protein expression. (**A**) Immunocytochemical staining of heme oxygenase, OAT-4, and DNA in 3D (left, 10x magnification) and 2D (right, 4x magnification) cultures. Treatments were stopped, and cells fixed at the dates noted above to avoid excessive loss of cell sample for later analyses. Cisplatin (6.4, 64 µM) and gentamicin (600 µM) were highly nephrotoxic, while a lower concentration of gentamicin (200 µM) and cadmium (0.05, 0.5 µM) demonstrated lower toxicities and were carried out to the full 21 days of exposure. Protein expression of (**B**) heme oxygenase and (**C**) OAT-4 are shown as percentage of field positively stained relative to vehicle controls. (*Significant change from relative vehicle, P < 0.05, 2-tailed t-test).

In these experiments (Fig. 5A), proximal tubules and 2D cultures without treatment showed confluent monolayers with intermittent expression of human organic anion transporter 4 (*OAT4*), an apical organic anion/dicarboxylate exchanger in the renal proximal tubules. Severe cytotoxicity was elicited by cisplatin and gentamicin in both 2D and 3D cultures. The experiments were terminated for 2D wells and 3D device treated with the highest concentration of cisplatin tested (64 μM) only after 3 days, while the experiment was terminated for 6.4 μM concentration after 12 days. Gentamicin cultures were maintained for 12 days (600 μM) or full 21 days (200 μM). Cadmium-treated cells were maintained for up to 21 days at both concentrations tested (0.05 and 0.5 μM). KIM-1 and LDH levels were evaluated in the cell culture media/perfusate (2D n = 5; 3D n = 3). KIM-1 levels did not increase by either treatment; to the contrary, the levels of KIM-1 were falling over time reflecting progressive loss of cell viability in 2D and 3D cultures (Supplemental Fig. 1). With respect to LDH, a similar pattern was observed, with an observed initial peak in secretion resulting from a few of the compound exposures (Supplemental Figs 2–3).

At the termination of each treatment/dose group, the cultures were fixed, fluorescently tagged for HO1 and OAT4 (Fig. 5A,B) and images quantified (2D n ≥ 3; 3D n ≥ 1). In 2D cultures, cisplatin significantly increased the expression of HO1 and OAT4 relative to controls, cadmium decreased the expression of HO1 at 0.05 μM, and gentamicin had no effect. Similar patterns were observed in 3D cultures, a large increase in HO1 and OAT4 was observed in cultures treated with 0.5 μM cadmium, and that 600 μM gentamicin appeared to increase expression of OAT4 in these cells; however, due to the lower throughput of the 3D MPS no replicate experiments were performed for the imaging phenotypes.

**Figure 6:**
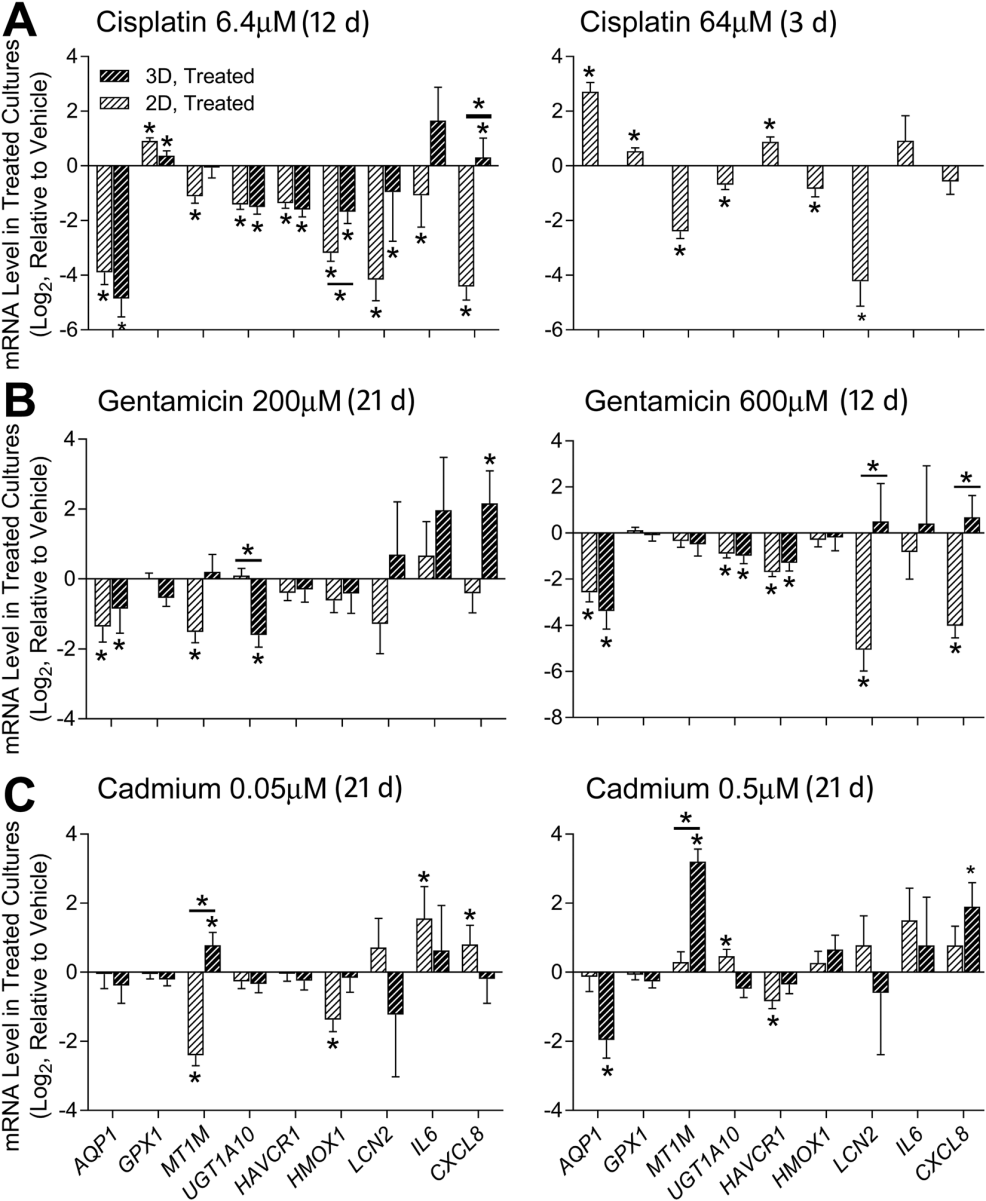
Effect of selected nephrotoxicants on 2D and 3D Lonza RPTEC gene expression. Expression of transporters and metabolic markers (AQP1, GPX1, MT1M and UGT1A10) and acute kidney injury markers (HACVR1, HMOX1, LXN2, IL6, CXCL8) is shown as mRNA level relative to vehicle treatments for 2D and 3D Lonza cultures after exposure to (**A**) Cisplatin, (**B**) Gentamicin, and (**C**) Cadmium for the duration shown. Differentially expressed genes (relative to controls) are marked with asterisks, and significant differences between 2D and 3D cultures are shown with bars. Analysis of cisplatin at 64 µM in 3D culture was not possible due to a lack of cell material present after treatment (*P < 0.05, 2-tailed t-test).

In addition, cell lysates were collected at each treatment’s respective termination and gene expression of kidney function (*AQP1*, *MT1M*, and *UGT1A10*) as well as toxicity (*HAVCR1*, *HMOX1*, *LCN2*, *IL6*, and *CXCL8*) markers was evaluated using targeted transcriptomics (Fig. 6). Gene expression patterns were similar between 2D and 3D cultures in response to cisplatin (6.4 μM) and gentamicin (both concentrations). Most of the effects of cisplatin and gentamicin on gene expression were downregulation of expression, concordant with severe toxicity. For cadmium-treated cultures, 2D and 3D cultures showed a discordant response, most pronounced in the upregulation of metallothionein 1M (*MT1M*) in 3D cultures, but down-regulation in 2D cultures.

## Discussion

This study’s principal goal was to use a case study of the technology transfer of the proximal tubule tissue chip model^14,15^ to an independent laboratory as the means to identify most critical elements that may impede the translation of MPS technology from a developer to an end-user. To achieve this goal, we compared critical endpoints from the original studies of the proximal tubule tissue chip model^14,15,27^ such as long-term viability of 3D cultures, ammoniagenesis, vitamin D metabolism, and nephrotoxic responses to polymyxin B to the outcome of the experiments performed at an independent laboratory. Beyond the more technical challenge of replicating the complex MPS technology, such as assembly and perfusion of a complex microfluidics device and repeating the experiments detailed above, we also evaluated several RPTEC sources, compared conventional 2D culture conditions in 384-well plates to the MPS, and investigated the effects of additional kidney-toxic compounds cisplatin^29^, gentamicin^30^, and cadmium^31^.

The overall rationale for this study was a well-acknowledged challenge with moving promising yet complex MPS from the developer labs to the end-users^1,9,10^. While rapid development of novel assays and technologies that have potential applications in biomedical research, drug development, and chemical safety evaluation is uniformly welcomed^1,9,32^, these assays and tools are usually not immediately useable in regulatory contexts. Before new assays are used in decision making, their relevance, reliability, and fitness for purpose should be established and documented. Such characterization of assays has evolved into elaborate processes that are commonly referred to as “validation of alternative methods”^13^. Formal mechanisms for validation have been established in many countries, including international standardization of validation methods^12^. In general, validation is a process which establishes and documents the reliability and relevance of a new method or assay for a defined purpose. The term reliability refers to the reproducibility of the method within and between laboratories when performed using the same protocol. The term relevance is meant to ensure that the outcome of the test is meaningful and useful for a particular purpose. In the context of this study, the relevance of the proximal tubule MPS was established by the developer’s laboratory^14,15^ and then key elements of the physiological and toxicological outcomes were tested for replication herein. While we used the same devices and protocols, and evaluated same or similar endpoints, we could not use the same cells, because the original primary cells were exhausted. Therefore, below we provide a detailed interpretation of the successes and challenges with technology transfer of this MPS, as well as discuss how these observations can be generalized to the field of tissue chip development.

First, we conclude that while the technology transfer and our ability to maintain long-term viability of cells in the MPS device were a success, the most striking difference with the original studies were observed because of the difference in sourcing of RPTECs. Specifically, even though Lonza cells maintained a healthy lumen in the device under flow for up to 24 days, their function differed from the primary RPTEC cell line in many key parameters (e.g., baseline shedding of KIM-1, vitamin D metabolism, etc.).

KIM-1 is a proximal tubule marker that has been linked to acute tubular injury as well as cellular repair and proliferation^33^. It is a qualified *in vivo* biomarker of kidney injury and is widely used in experimental animals and humans^34^. While levels of KIM-1 were similar while comparing 2D and 3D cultures within a single cell source, when comparing between cell sources, we observed a 20-fold increase in baseline KIM-1 in the Lonza cells. KIM-1 is often used as a proliferative marker and the basal proliferation rate of HIM-31 cells was much lower than that of Lonza cells. These results indicate that considerable differences between cell sources for RPTECs, and possible inter-individual differences, may exist and can affect the outcomes of the experiments in the tissue chip.

The proximal tubule is a major site of vitamin D bioactivation, where the prohormone 25-hydroxyvitamin D3 is metabolically converted to the active metabolite 1α,25-dihydroxyvitamin D3 (calcitriol)^35^. In the presence of circulating calcitriol, metabolism shifts to favor the formation of 24,25-dihydroxyvitamin D3 (24,25-(OH)_2_ vitamin D3), an inactive metabolite fated for excretion, via CYP24A1^36,37^. The original MPS development study^14^ demonstrated that when RPTECs were cultured within the microphysiological system under shear, cells metabolized calcidiol to quantifiable levels of metabolites calcitriol, 24,25-(OH)_2_ vitamin D3, and 4β,25-(OH)_2_ vitamin D3. Additionally, it was shown that in the presence of exogenous calcitriol, 24, 25-(OH)_2_ formation clearance was increased, accompanied by an increase in expression of *CYP24A1*. In our study, formation clearance of 24, 25-(OH)_2_ vitamin D3 significantly increased in 3D HIM-31 cell cultures; however, it was unaffected in Lonza cell 3D cultures. In addition, HIM-31 cells showed an increase in CYP24A1 expression, which was nearly identical to the 54-fold increase reported by Weber *et al*.^14^. Lonza cells responded with strong induction of *CYP24A1*, albeit it was about half of that seen in HIM-31 cells. Regardless, the results in 3D culture are consistent with *in vivo* observations where an accelerated calcitriol clearance rate is observed following administration of 24, 25-(OH)_2_ vitamin D3^38^.

Ammoniagenesis is another crucial physiological function of the proximal tubule. In response to acidosis, the catabolism of glutamine leads to the net formation of two NH_4_^+^ and HCO_3_^−^ ions per glutamine, balancing the acidic environment of the cells^39^. Weber *et al*.^14^ demonstrated that when RPTECs were exposed to a decrease in luminal pH within their culturing platform, a 3-fold increase in effluent ammonia concentration was observed. However, the results of our study did not show the same effect. Instead, a slow decrease in ammonia concentration was observed regardless of cell source. This discrepancy in results between testing sites may in part be due to cell sourcing. Although HIM-31 cells were directly sourced from the developer lab, these primary cells were isolated from a donor different from that reported in Weber *et al*.^14^, once again highlighting the need for a commercially available cell source. In addition to measuring ammonia concentration in the effluent, transcriptomic analyses showed that in HIM-31 expression of *HAVCR1* and *SLC7A11* was increased, and *GLUD1* decreased, indicative of potential cellular injury in response to the altered environment, which may in turn explain the lower baseline ammonia concentration in the effluent. In cultures with Lonza cells, however, *HAVCR1* expression was unaffected, and expression of *SLC7A11* and *GLUD1* was increased; however, this did not translate to a change in ammonia generation in response to altered luminal pH.

Second, because most MPS development studies do not include a comparison with conventional *in vitro* models, we also carried out parallel experiments with 2D cell cultures. Indeed, human RPTECs are an already well-established *in vitro* model for assessment of cytotoxic effects of nephrotoxic agents^40^. Recent studies showed that fluid sheer stress can be applied to 2D cultures of RPTECs and improve the physiological characteristics of the *in vitro* model^41^; however, the throughput of this model is low, as it requires a 12-well culture format. Our 2D studies were aimed at high-throughput experiments to enable queries of multiple compounds, concentrations and replicates; hence, we chose to conduct experiments in 2D without flow to simulate a test system that would be most commonly used in absence of MPS.

While 2D cell cultures are higher throughput and more accessible than tissue culture platforms, it is important to ensure the physiological relevance of these cells as compared to tissue chips and whole tissues, and determine what level of complexity is necessary to capture specific endpoints. While both HIM-31 and Lonza cells were intact in 2D over 24 days, morphology of cells that were grown in 3D was more consistent with that *in vivo*, an effect of shear stress^42–45^. Cell elongation and self-organization is essential to maintain cell differentiation and polarization of RPTECs. In addition, while 2D and 3D cultures exhibited similar patterns of LDH activity within the first 14 days of treatment, after that cells grown in 2D began to show elevated levels of LDH activity where 3D cultures remained consistent. Baseline gene expression was also investigated, and it was found that while there were a few differences between 2D and 3D cultures, basal gene expression was similar between cell sources and culture conditions, and close to the gene expression profile of human kidney cortex from GTEx^22,23^. From this view, it would appear that untreated 2D and 3D cultures behave similarly. However, in the vitamin D study, 2D cultures of both HIM-31 and Lonza cells showed a significant decrease in the net formation of the inactive metabolite 24,25-(OH)_2_ vitamin D3. Neither was there an effect on *CYP24A1* expression in 2D cultures. It is possible that these cells have a low ceiling of maximal induction, and that the calcidiol vehicle (which can have low levels of VDR affinity^46^) is sufficient to achieve this maximal induction in 2D. We propose that 3D culturing conditions may have restored some level of inducibility in these cells due to the more realistic microenvironment, however this will require further investigation. These data show that 2D cultures have considerable limitations for the study of complex biological phenomena, consistent with observations of rapid loss of advanced function and differentiation of multiple cell types grown for extended periods in 2D^47–49^.

Third, we investigated responses of RPTECs in 2D or 3D to several prototypical nephrotoxic compounds. RPTEC cultures grown in both 2D and 3D were initially challenged with polymyxin B, an antibiotic with known toxicity to the human proximal tubule^50^. Previous *in vitro* studies with proximal tubule cell lines have demonstrated extensive tubular injury and apoptosis by polymyxin B^27,51,52^. In this study, HIM-31 and Lonza cells grown in both 2D and 3D culture were exposed to 50 μM polymyxin B for a 48 hour period. These studies indicate that HIM-31 cells in a 3D MPS were more robust and resistant to the cell death from polymyxin B, but that both 2D and 3D grown HIM-31 cells exhibit “realistic” nephrotoxic responses through increases in stress markers lipocalin^53^, heme oxygenase^54^, fibronectin^55^, and a xenobiotic metabolism enzyme UDP-glucuronosyltransferase^56^. Previous studies by Weber *et al*.^27^ were consistent with these results, indicating a significant increase in ROS production, and cell death only at concentrations >100 µM after a 24 hour exposure. Lonza cells were much more sensitive to polymyxin B as complete cell loss was observed at 48 hrs after treatment in both 2D and 3D cultures.

Additional studies were carried out with cisplatin, gentamicin, and cadmium, which are well characterized nephrotoxic agents. Cisplatin and cadmium concentrations were selected to bracket human C_max_ values; however, gentamicin was already contained within the formulation of the cell culture media at near human C_max_ levels, so to challenge the cells, higher concentrations were selected for this compound. Cultures were exposed to these compounds until significant tubular disruption was observed, up to 21 days and we observed cytotoxicity at concentrations and time points similar between 2D and 3D culture conditions. At each treatment’s respective termination endpoint, we investigated expression of heme-oxygenase-1 (*HO1*) and organic anion transporter-4 (*OAT4*). In 2D culture, cisplatin significantly increased the expression of *HO1* and *OAT4* relative to controls, and cadmium decreased the expression of *HO1* at the lower concentration. Similar patterns were observed in 3D culture; however, due to low throughput and lack of replicates, significance of the effect cannot be established. However, a large increase in *HO1* and *OAT4* was seen in the 3D cultures treated with the higher concentration of cadmium, indicating oxidative stress, and an increase in transport in response to the presence of cadmium. Though no studies investigated the OAT-mediated uptake of cadmium, an increase in basolateral uptake was observed after the co-administration of cadmium with cysteine and glutathione in rat kidneys^57^. Gentamicin also notably increased *OAT4* in cells cultured in 3D conditions, which could potentially explain the accumulation of aminoglycosides in the kidney often seen after treatment with these compounds^58^. Similarly to the studies with polymyxin B, KIM-1 concentration in the cell culture media or effluent was not indicative of the toxicity as the levels of KIM-1 decreased as cell death increased over time. LDH concentration, on the other hand, was significantly increased relative to controls in many of the treatments prior to falling off along with cell viability, indicating that it may be a more suitable biomarker for *in vitro* nephrotoxicity studies^59^.

## Conclusions

This study tested the transferability of a complex MPS, a human renal proximal tubule chip. We conclude that while the overall transferability of this MPS was a success, the reproducibility with the original report was greatly dependent on the cell source. Each MPS consists of at least two distinct and critically important components, the actual device and the cells. Together, they create the MPS and cannot be uncoupled when either transferability or reproducibility are considered. Our data indicate that while freshly isolated RPTECs may be more “physiological” in their responses and function, commercial cells may be the only option for transfer of the technology to most of the end-users and increasing its throughput. Our study demonstrates critical importance of developing microphysiological platforms using renewable cell sources and makes a compelling case that every MPS must be joined with a commercially available well-characterized cell population, a path that will ensure reproducibility of the MPS technology. In addition, while 2D and 3D cultures may initially appear to exhibit many of the same responses from an apical view, the use of 3D cultures with media flow leads to a more realistic recapitulation of complex functions and responses of the proximal kidney tubule. Therefore, it is important to balance the complexity and throughput of 2D and 3D culture techniques against their physiological relevance when choosing a “fit for purpose” experimental *in vitro* model.

## Electronic supplementary material

Supplemental Figures

## Data Availability

All data collected during the current study are available from the corresponding author upon reasonable request.
