# Supplementary material for: Technology Transfer of the Microphysiological Systems: A Case Study of the Human Proximal Tubule Tissue Chip
Source: Sci Rep. 2018 Oct 5;8:14882. doi: 10.1038/s41598-018-33099-2 (PMC6173737; doi:10.1038/s41598-018-33099-2)

Technology Transfer of the Microphysiological Systems:

A Case Study of the Human Proximal Tubule Tissue Chip

Courtney Sakolish[a], Elijah J. Weber[b], Edward J. Kelly[b], Jonathan Himmelfarb[c], Roula Mouneimne[a], Fabian A. Grimm[a], John S. House[d], Terry Wade[e], Arum Han[f,g], Weihsueh A. Chiu[a], Ivan Rusyn[a,*]

[a]Department of Veterinary Integrative Biosciences, Texas A&M University, College Station, TX

[b]Department of Pharmaceutics, University of Washington, Seattle, WA

[c]Division of Nephrology, University of Washington Kidney Research Institute, Seattle, WA

[d]Bioinformatics Research Center, North Carolina State University, Raleigh, NC

[e]Geochemical and Environmental Research Group, Texas A&M University, College Station, TX

Departments of [f]Electrical and Computer Engineering, and [g]Biomedical Engineering, Texas A&M University, College Station, TX

***Corresponding author:**

Ivan Rusyn, MD, PhD; Department of Veterinary Integrative Biosciences, 4458 TAMU, Texas A&M University, College Station, TX; 979-458-9866; irusyn@tamu.edu

Supplemental Figure 1. Effect of selected nephrotoxicants on 2D and 3D Lonza RPTEC KIM-1 secretion. KIM-1 secretion was measured over each exposure period up to day 21 in RPTECs that were treated with (A) cisplatin, (B) gentamicin, or (C) cadmium. Baseline (control) KIM-1 secretion remains relatively constant in 2D and 3D for the first 10 days of culture, however, longer culture leads to an increasing trend in 3D, and an overall decrease in secretion in 2D over time. Significant differences between treatment and associated controls are indicated. KIM-1 secretion may not be a sensitive enough marker for acute nephrotoxicity in this tissue culture model, as no elevated levels are seems here after drug treatment. Rather, KIM-1 appears to follow along with cell viability, where lower levels are associated with fewer viable cells within devices or wells. (*P<0.05, 2-tailed t-test)

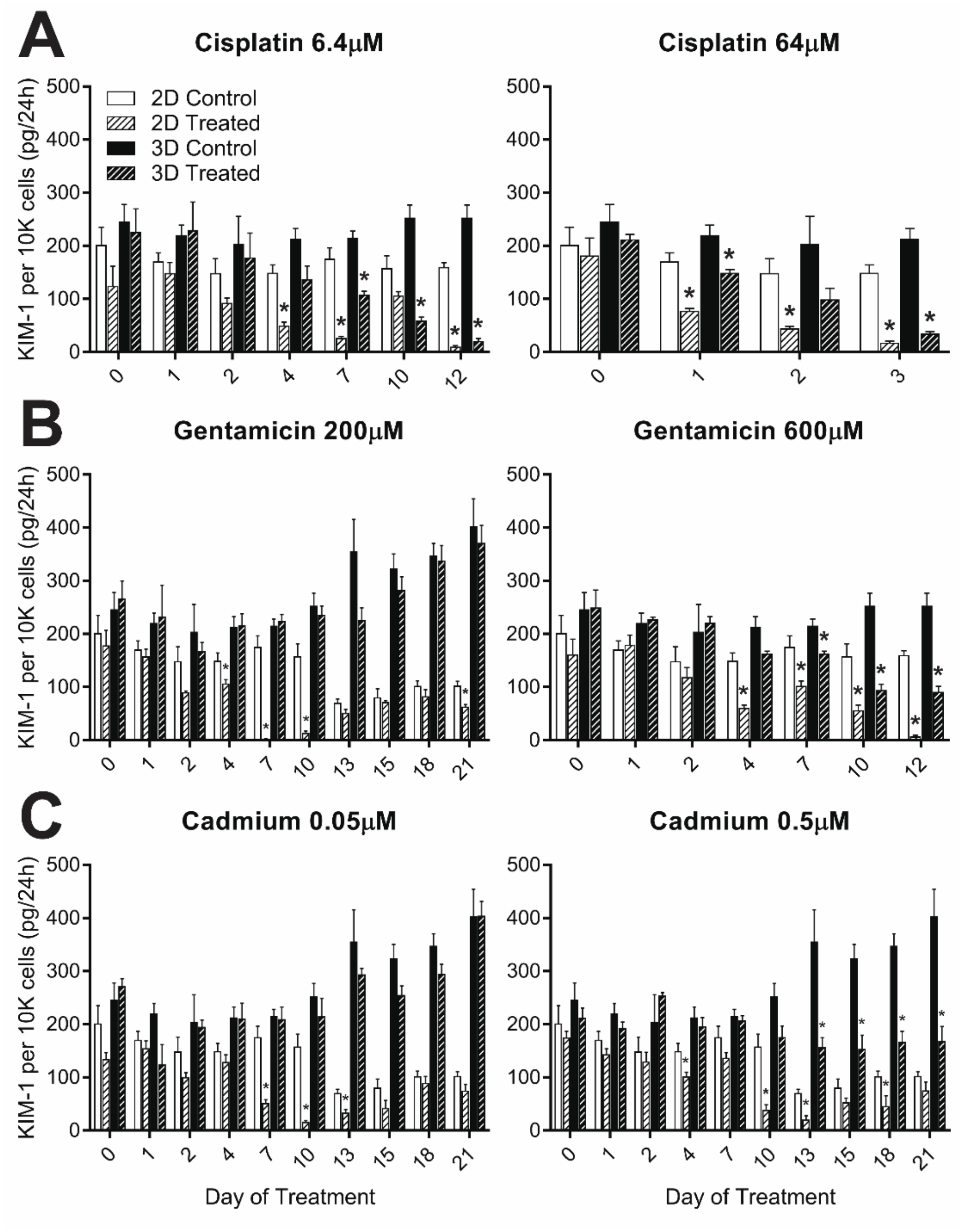

Supplemental Figure 2. LDH secretion in (A) HIM-31 and (B) Lonza RPTECs cultured in 2D and 3D. LDH secretion is elevated in 3D cultures over the first 2 weeks of culture, however a significant increase in levels is observed over long term culture (up to day 24) in 2D Lonza RPTECs. Significant differences between 2D and 3D are indicated above. (*P<0.05, 2-tailed t-test).

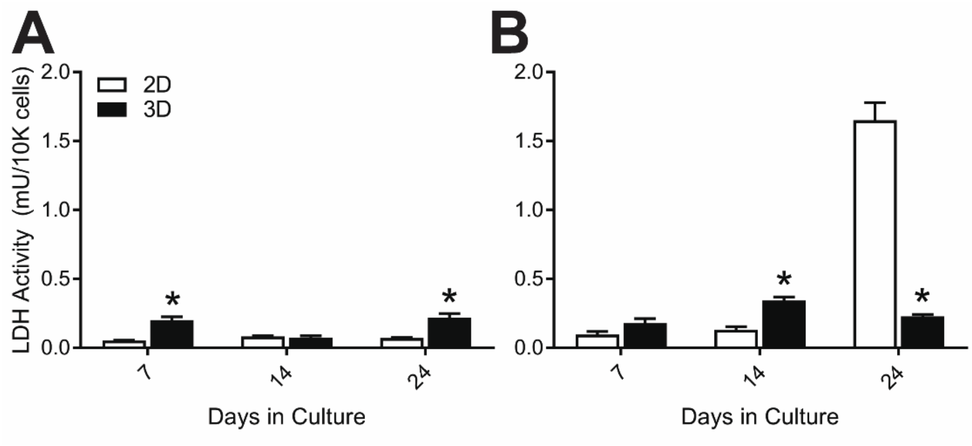

Supplemental Figure 3. Effect of selected nephrotoxicants on 2D and 3D Lonza RPTEC LDH activity. LDH activity was measured over each exposure period up to day 21 in RPTECs that were treated with (A) cisplatin, (B) gentamicin, or (C) cadmium. Baseline (control) LDH activity remains relatively constant in 2D and 3D for the first 13 days of culture, however, longer culture leads to an increasing trend in 2D, with activity in 3D cultures remaining constant. LDH is initially elevated in a few of the exposure conditions—however, shows similar patterns to KIM-1, mainly following along with cell viability. Significant differences between treatment and associated controls are indicated. (*P<0.05, 2-tailed t-test).

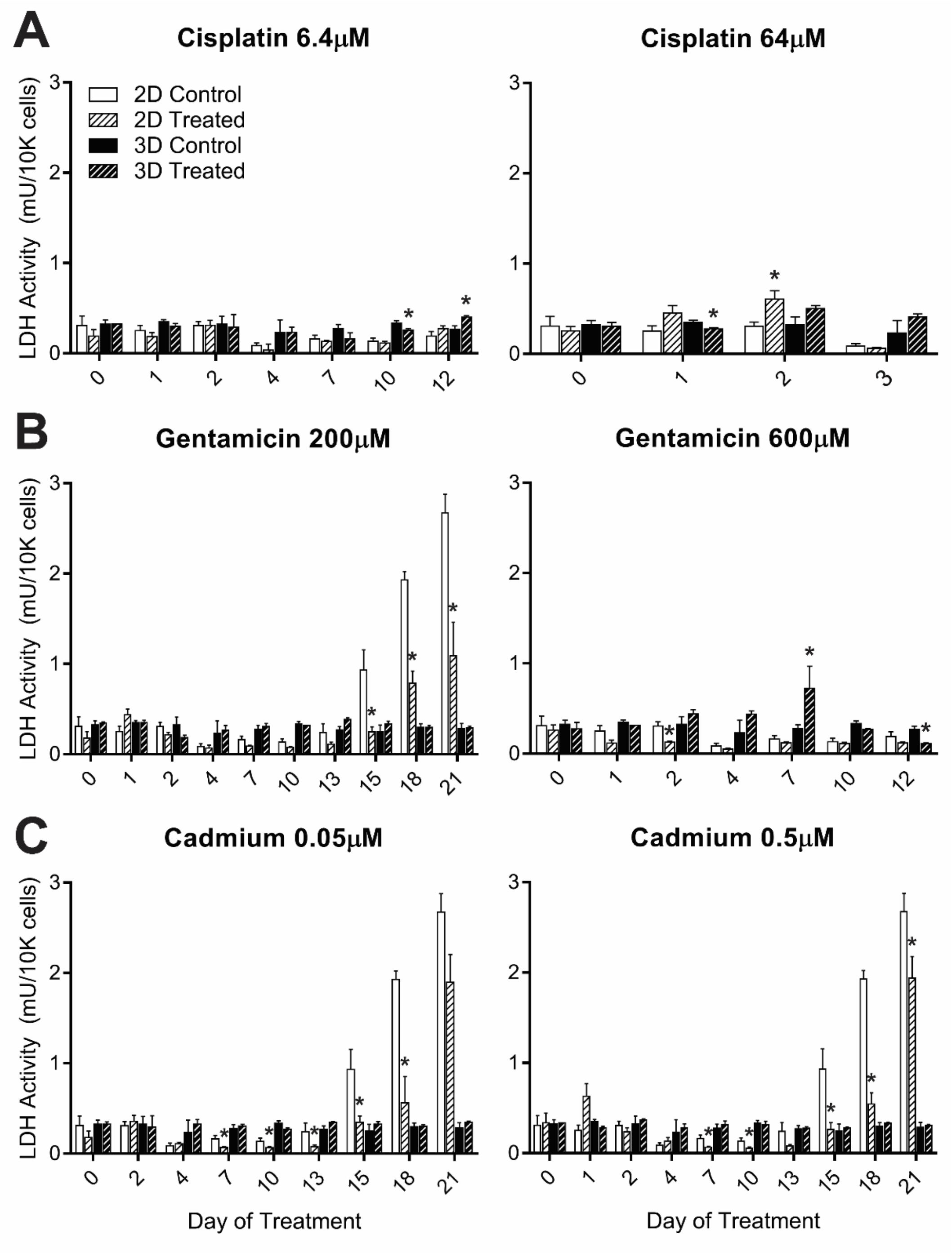

Supplement: Supplementary file 1 — Supplemental Figures [file 41598_2018_33099_MOESM1_ESM.pdf]
